# Shape Memory Polymer Foams with Phenolic Acid-Based Antioxidant Properties

**DOI:** 10.3390/antiox11061105

**Published:** 2022-06-01

**Authors:** Changling Du, David Anthony Fikhman, Mary Beth Browning Monroe

**Affiliations:** Biomedical and Chemical Engineering, BioInspired Institute, Syracuse University, Syracuse, NY 13244, USA; cdu108@syr.edu (C.D.); dafikhma@syr.edu (D.A.F.)

**Keywords:** phenolic acids, shape memory polymer, polyurethane, oxidative degradation

## Abstract

Phenolic acids (PAs) are natural antioxidant agents in the plant kingdom that are part of the human diet. The introduction of naturally occurring PAs into the network of synthetic shape memory polymer (SMP) polyurethane (PU) foams during foam fabrication can impart antioxidant properties to the resulting scaffolds. In previous work, PA-containing SMP foams were synthesized to provide materials that retained the desirable shape memory properties of SMP PU foams with additional antimicrobial properties that were derived from PAs. Here, we explore the impact of PA incorporation on SMP foam antioxidant properties. We investigated the antioxidant effects of PA-containing SMP foams in terms of in vitro oxidative degradation resistance and cellular antioxidant activity. The PA foams showed surprising variability; p-coumaric acid (PCA)-based SMP foams exhibited the most potent antioxidant properties in terms of slowing oxidative degradation in H_2_O_2_. However, PCA foams did not effectively reduce reactive oxygen species (ROS) in short-term cellular assays. Vanillic acid (VA)- and ferulic acid (FA)-based SMP foams slowed oxidative degradation in H_2_O_2_ to lesser extents than the PCA foams, but they demonstrated higher capabilities for scavenging ROS to alter cellular activity. All PA foams exhibited a continuous release of PAs over two weeks. Based on these results, we hypothesize that PAs must be released from SMP foams to provide adequate antioxidant properties; slower release may enable higher resistance to long-term oxidative degradation, and faster release may result in higher cellular antioxidant effects. Overall, PCA, VA, and FA foams provide a new tool for tuning oxidative degradation rates and extending potential foam lifetime in the wound. VA and FA foams induced cellular antioxidant activity that could help promote wound healing by scavenging ROS and protecting cells. This work could contribute a wound dressing material that safely releases antimicrobial and antioxidant PAs into the wound at a continuous rate to ideally improve healing outcomes. Furthermore, this methodology could be applied to other oxidatively degradable biomaterial systems to enhance control over degradation rates and to provide multifunctional scaffolds for healing.

## 1. Introduction

Wound healing involves the synergistic action of multiple tissue types with different growth factors, cytokines, and hormones [1]. In this complex process, reactive oxygen species (ROS) play an important role [2]; ROS is a broad term for chemical species that contain oxygen radicals [3]. The ROS family includes superoxide anions (O_2_^•−^), peroxides (O_2_^•−2^), hydrogen peroxide (H_2_O_2_), hydroxyl radicals (^•^OH), and hydroxyl ions (OH^−^) [4]. Typical cellular metabolic activity includes continuous production of ROS during mitochondrial oxidative metabolism, which contributes to the regulation of cell growth, adhesion, differentiation, senescence, and apoptosis [5]. Another major source of ROS is nicotinamide adenine dinucleotide phosphate (NADPH) oxidase in the inflammatory response. Inflammation is a defensive immune response against foreign substances or tissue damage. Thus, when the immune system is activated, phagocytes produce ROS at low concentrations to destroy foreign substances, such as bacteria, at sites of inflammation.

Healthy cells have antioxidant defenses to scavenge ROS independently in cases where ROS levels are too high; however, excess ROS production from the inflammatory response can exceed the cellular endogenous antioxidant capacity, resulting in oxidative stress in wound tissue. If these defense mechanisms remain activated, acute inflammation can turn into chronic inflammation [6]. Previous work has shown that NADPH oxidase produces excess ROS, including highly reactive superoxide radical anions, due to stimulation by tumor necrosis factor-α [7], which can result in cell damage in the wound or inflamed tissue [8]. This phenomenon is called “respiratory burst”. ROS is also a significant cause of wound fibrosis and scar formation and can aggravate wound tissue damage [9]. A characteristic of chronic wounds and slow-to-heal wounds in the elderly is the excess accumulation of ROS due to the failure of endogenous ROS scavenging defenses [8] Antioxidant wound dressings with ROS scavenging ability can therefore play a crucial role in healing and have been reported to accelerate wound closure [10]. To that end, in this work antioxidant phenolic acids (PAs) were incorporated into a shape memory polymer (SMP) foam network to provide a wound healing scaffold with antioxidant properties.

Plant phenolics are important dietary antioxidants and are the broadest secondary metabolites of plants [11]. Phenolics are used in the dietary and medical fields due to their excellent antimicrobial [12,13,14,15], anti-inflammatory [16,17,18], anticancer [19,20,21,22], antiallergy [23,24], and antioxidant [25,26,27,28,29] properties. One subclass of plant phenolics is PAs, which contain a carboxylic acid group. PAs can provide antioxidant properties through three potential mechanisms: (1) ROS scavenging from the donation of their H atoms [30], (2) inhibition of enzymes or chelating agents that can generate ROS, and (3) improving cellular antioxidant defenses, with (1) ROS scavenging being the most commonly described [19,31]. Multiple PAs have been reported to reduce cellular oxidative damage through ROS scavenging, including caffeic acid, cinnamic acid, ferulic acid, gallic acid, syringic acid, vanillic acid, and protocatechuic acid [32]. In our previous work, multiple PAs also demonstrated H_2_O_2_ scavenging ability [33].

A scaffold for controlled release of PAs and wound filling is important for healing. SMPs are ‘smart’ materials that can maintain a temporary shape and recover back to a permanent shape under stimulation, such as a change in temperature. SMP materials have been widely used in biomedical applications due to the benefits of their unique shape memory properties in drug delivery, bone tissue engineering, and cardiovascular applications [34]. Polyurethane (PU) SMP foams are a specific class of SMPs used as biomaterials. For example, temperature-response PU SMP foams can be used as a hemostatic agent to stop bleeding in wounds [35,36]. These PU foams are oxidatively degraded through cleavage of the C-N groups in the polyols (*N*,*N*,*N*′,*N*′-tetrakis(2-hydroxypropyl) ethylene-diamine (HPED) and triethanolamine (TEA)) used in their synthesis, leading to fragmentation. The study of oxidative degradation of PU SMP foams is important for their potential use as long-term biomaterial implants, and control over degradation enables controlled release of incorporated bioactive agents, such as PAs.

To increase biomaterial scaffold lifetimes, many approaches have been taken to improve oxidative resistance of polyurethane materials. These include incorporation of antioxidants, which has been effective at slowing down the rate of oxidative degradation. For example, antioxidant poly(urethane urea) microparticles were previously incorporated into porous SMP foams. The microparticles increased the biostability of the SMPs in accelerated oxidative degradation testing by 25% [37]. Antioxidant delivery has been used in various forms in biomaterials, such as SiO_2_-based nanoparticles (NPs) [38,39,40], silver-based NPs [41,42], and polymeric NPs [43,44,45,46], to impart antioxidant properties [47] for healing purposes.

In our previous research [33,48], we analyzed the antimicrobial and antioxidant properties of 10 PAs, from which three PAs with high antioxidant properties were selected: p-coumaric acid (PCA), vanillic acid (VA), and ferulic acid (FA). We successfully incorporated these PAs into the PU SMP foam network to provide PA-containing SMP foams. These PA SMP foams exhibit excellent antimicrobial, thermal, shape memory properties, cell compatibility, and blood compatibility, but only moderate H_2_O_2_ scavenging. In this work, we further investigated the effect of PA-containing SMP foams on antioxidant activity in terms of oxidative degradation resistance and cellular antioxidant ability.

## 2. Materials and Methods

### 2.1. Materials

All chemicals were purchased from Fisher Scientific (Pittsburgh, PA, USA). Caco-2 cells were purchased from ATCC (Manassas, VA, USA). The 2′,7′ –dichlorofluorescin diacetate (DCFDA/H_2_DCFDA)–Cellular ROS Assay Kit (Abcam) was purchased from Abcam (Cambridge, UK).

### 2.2. PA Foam Synthesis

SMP foams were synthesized with PCA, VA, and FA using a previously described method [45]. Briefly, PAs, HPED, and TEA were mixed at a 1:7:2 molar ratio of COOH (PAs) and OH (HPED and TEA) groups. The COOH/OH components were reacted with excess hexamethylene diisocyanate (HDI) (0.35 mole COOH + OH: 1 mole isocyanate (NCO)) in tetrahydrofuran (THF) at 50 °C for 48 h to form the NCO pre-polymer. A second COOH/OH solution was prepared with PAs, HPED, and TEA in the same molar ratio in tetrahydrofuran (THF) in an amount required to achieve a final 1:1 ratio of COOH (from PAs) + OH (from TEA and HPED): NCO. The OH solution was mixed with T-131 and BL-22 catalysts at 0.5 and 1.1 wt%, respectively, EPH190 surfactant at 9 wt%, and deionized water as a chemical blowing agent using a speed mixer (FlackTek, Inc., Landrum, SC, USA). T-131, BL-22, and EPH190 were all generously provided by DOW, Inc. (Midland, MI, USA). The OH solution was reacted with the NCO pre-polymer at 50 °C to form a PA-containing SMP foam [35]. A control foam was prepared using the same protocol without the addition of PAs (HPED:TEA = 7:3). The resulting foams were washed twice with 70% ethanol and DI water and then dried in a 50 °C vacuum oven to remove residual chemicals.

### 2.3. Oxidative Degradation

ROS-induced oxidative degradation was characterized using a modified version of a previously described method [49]. Cylindrical PA foam samples were cut to 8 mm diameter and 10 mm height (*n* = 8) using a biopsy punch (Sklar Instruments, West Chester, PA, USA). All samples were weighed and then placed in separate sealed vials with 15 mL of 20% H_2_O_2_. The vials were placed in an incubator at 37 °C. Sample foams were analyzed in terms of mass remaining every 2 days and glass transition temperature (T_g_), surface chemistry, and pore structure every 4 days, according to the below protocols. Before analysis, samples were washed with 70% ethanol and placed in a 50 °C vacuum oven overnight to remove 20% H_2_O_2_ and dry. After measurement, the samples were returned to the sealed vials with fresh 20% H_2_O_2_ solution in the 37 °C incubator.

#### 2.3.1. Mass Loss

Dried foam samples (*n* = 4) were weighed using a scale every 2 days.

#### 2.3.2. Pore Structure

Foam pore structure was analyzed by Jeol NeoScope JCM-5000 Scanning Electron Microscope (SEM, Nikon Instruments, Inc., Melville, NY, USA). A 1 mm thick sample was cut from the dried 8 mm diameter cylinder and fixed with double-sided tape onto a SEM sample holder. Then, the foam samples were coated with Au for 45 s using a sputter coater (Denton Vacuum Desk II, Moorestown, NJ, USA) and imaged.

#### 2.3.3. Glass Transition Temperature

The Tg of dried foams was analyzed using a Q-200 differential scanning calorimeter (DSC, TA Instruments, Inc., New Castle, DE, USA). A sample (3–5 mg) was cut from the cylinder and dried under vacuum at 50 °C overnight. The dried foam sample was then placed into a Tzero aluminum pan with an aluminum lid (DSC Consumables, Inc., Austin, MN). Foam samples were subjected to the following program: (1) equilibrated for 2 min at −40 °C; (2) heated to 120 °C at 10 °C per minute; (3) equilibrated for 2 min at 120 °C; (4) cooled to −40 °C at −10 °C per minute; (5) equilibrated for 2 min at −40 °C; and (6) re-heated to 120 °C at 10 °C per minute in a second heating cycle. The foam Tg was analyzed as the endothermic inflection point of the DSC thermogram in the second heating cycle using TA instruments software (TA Instruments, Inc., New Castle, DE, USA) as previously described [36].

#### 2.3.4. Surface Chemistry

The surface chemistry of thin slices of dried foam samples was analyzed using attenuated total reflectance (ATR)-Fourier transform infrared (FTIR) spectroscopy (Nicolet i70 Spectrometer, Fisher Scientific, Waltham, MA, USA) at 0.8 cm^−1^ resolution.

### 2.4. PA Release from SMP Foams

Foam samples were placed in separate sealed vials with 15 mL of PBS in an incubator at 37 °C. A portion of the PBS solution (500 µL) was collected from each PA foam sample at 0.5, 1, 2, 4, 8, 24 h, and then every 24 h up to two weeks. The solutions were diluted in DMSO (PBS:DMSO = 3:2) and placed into a black walled cuvette to measure absorbance using a Cary 60 UV-vis spectrophotometer (Agilent Technologies, Santa Clara, CA, USA). The PA concentrations were quantified by comparison with PA standard curves. If sample solution concentrations were too high (maximum absorbance > 2), they were diluted 10 times using the 3: 2 PBS: DMSO solution. PA standard curves for PCA, VA, and FA solutions in 3: 2 PBS: DMSO were prepared by varying PA concentrations from 2 to 18 µg/mL.

### 2.5. Cell Culture

Human colon adenocarcinoma (Caco-2) cells were purchased from ATCC. Caco-2 cells were cultured in Minimum Essential Media (MEM, Gibco^TM^, Thermo Fisher Scientific, Waltham, MA, USA) with phenol red supplemented with 10% heat-inactivated fetal bovine serum (FBS, Gibco^TM^) and 1% penicillin-streptomycin (PS, Gibco^TM^) and maintained at 37 °C/5% CO_2_ incubator. Caco-2 cells were used between passages 2 and 6.

#### 2.5.1. Cellular Antioxidant Activity (CAA) Assay

A cellular antioxidant activity (CAA) assay was modified from previously described methods of Wolfe et al. [50] and Kellett et al. [51]. Foam samples were cut into cylinders (8 mm diameter, 2 mm height, *n* = 3), immersed in 1 mL 70% ethanol for sterilization, and then washed 3 times in sterile phosphate-buffered saline (PBS). A CAA assay was performed with Caco-2 cells using the DCFDA/H_2_DCFDA-Cellular ROS assay kit (Abcam, Cambridge, UK). Caco-2 cells were seeded at 6 × 10^4^ cells/well in a black tissue culture-treated 96-well plate (USA Scientific, Inc., Ocala, FL, USA) and incubated in 150 µL MEM with 10% FBS and 1% PS for 48 h at 37 °C/5% CO_2_. After 48 h, the media was removed, and the cells were washed with sterile PBS. Then, 50 µL of MEM/FBS/PS without phenol red and 50 µL of 20 µM DCFDA solution in PBS were added to each well. The cells were cultured in a dark incubator for 1 h at 37 °C.

Simultaneously, washed foam samples were incubated in a second 96-well plate with 150 µL of 0.01% H_2_O_2_ in Hank’s balanced salt solution (HBSS) buffer or 600 µM ABAP in HBSS buffer at 37 °C for 1 h. Control wells contained H_2_O_2_ or ABAP solutions without foam samples. The media was removed from the cells in the first 96-well plate, and the cells were washed with PBS to remove extracellular DCFDA reagents and ensure that only intracellular ROS was quantified. Then, 100 µL of the HBSS solutions were transferred from the sample-containing second 96-well plate onto the cells in the first well plate. This well plate was immediately placed into a plate reader (FLx800, Bio-Tek Instrument, Inc., Winooski, VT, USA). The kinetic fluorescence was read every 5 min for 1 h at an excitation wavelength of 485 nm and an emission wavelength of 528 nm. Care was taken to protect the black 96-well plate from light to prevent photolytic reaction of the DCFDA reagent in the experimental process.

#### 2.5.2. Quantitation of CAA

The effects of foam samples on cellular antioxidant properties were quantified by calculating the area under the curve (AUC) at each 5 min fluorescence measurement. The ROS control group (without any antioxidant reagents) was taken as the maximum intensity of oxidized fluorescence from DCF formation. The CAA unit was then measured using Equation (1):

(1) $$\mathrm{CAA}\;\mathrm{Unit}\;\left(\%\;\mathrm{reduction}\right)=\left(1-\frac{{\mathrm{AUC}}_{\mathrm{foam}\;\mathrm{sample}}}{{\mathrm{AUC}}_{\mathrm{ROS}\;\mathrm{control}}}\right)\times 100\%$$

where CAA Unit is cellular antioxidant activity units, AUC_foam smaple_ is the area under the curve of foam sample fluorescence measurements, and AUC_ROS control_ is the area under the curve of the positive control group fluorescence measurements. Higher antioxidant activity is associated with reduced fluorescence intensity compared with the ROS control group fluorescence.

#### 2.5.3. Cytocompatibility Assay

Cell viability of Caco-2 cells was confirmed using the Alamar Blue assay. All initial steps were consistent as described in Section 2.5.1 for the CAA assay. Then, instead of placing the first plate into the plate reader after transferring the solutions from the second 96-well plate, cells were incubated with sample solutions for 1 h at 37 °C. The solutions were removed, and the wells were washed with PBS. Cells were incubated with 100 µL of 10% Alamar Blue solution at 37 °C for 2 h. Positive control group wells contained MEM/FBS/PS media without DCFDA reagents and ROS generators (H_2_O_2_ and ABAP). The fluorescence intensity was read with excitation of 530 nm and emission of 590 nm using a plate reader. Cell viability was measured using Equation (2):

(2) $$\mathrm{Cell}\;\mathrm{Viability}\;\left(\%\right)=\frac{{\mathrm{OD}}_{\mathrm{sample}}}{{\mathrm{OD}}_{\mathrm{positive}\;\mathrm{control}}}\times 100\%$$

where OD_sample_ is the optical density of foam sample, and OD_positive control_ is the positive control described above.

### 2.6. Statistics

All statistical analysis was conducted using Microsoft Excel. Data were reported as mean ± standard deviation. Student’s *t*-tests were performed to determine the difference between PA foams and controls. Statistical significance was taken as *p* < 0.05.

## 3. Results and Discussion

### 3.1. In Vitro Oxidative Degradation

#### 3.1.1. Gravimetric Analysis

**Figure 1:**
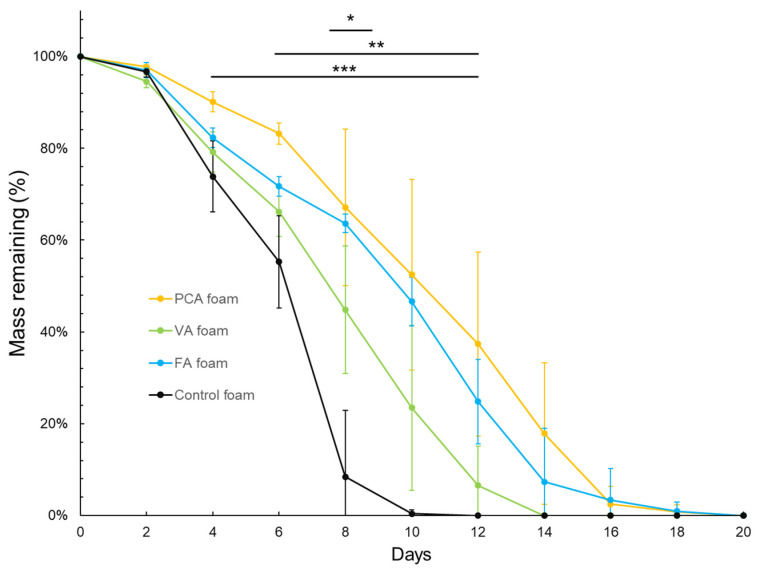
Gravimetric analysis of SMP foams in accelerated oxidative degradation media (20% H_2_O_2_) over 20 days. *n* = 4, mean ± standard deviation displayed, * *p* < 0.05 between VA foam and control foam, ** *p* < 0.05 between FA foam and control foam, *** *p* < 0.05 between PCA foam and control foam.

Gravimetric analysis of the SMP foams in an accelerated oxidative degradation media (20% H_2_O_2_) at 37 °C over 20 days is shown in Figure 1. The PA foams demonstrate a resistance to oxidative degradation compared with control foams without PAs. There was no significant difference in the masses of the tested SMP foam formulations during the first two days of degradation. After two days, the mass of the control foam was reduced by 20% every two days until day 6. Then, more rapid degradation was observed with a mass decrease from 55% to 8% over days 6 and 8. The control foam was fully degraded by the 10th day. In contrast, the degradation rate of the PCA foam was slower from day 4 to day 6. On the 6th day, when the control foam was almost 50% degraded, the PCA foam still maintained 83% of its mass. After six days, the degradation rate of the PCA foam started to accelerate, but this formulation retained the slowest degradation rate of all foam samples until day 16. PCA foam degradation was then comparable to FA foam degradation between days 16 and 20, at which point PCA foams were completely degraded. FA foams showed a relatively uniform degradation rate followed by accelerated mass loss rates on the 10th day and complete degradation at 20 days. VA foams degraded at an intermediate rate, with complete degradation at 14 days.

Notably, PCA and FA foam time to 100% mass loss under accelerated conditions was 20 days, double the time of the control foam. Overall, the degradation time of PA-containing foams is extended by a factor of 1.4–2× compared with control foams. In previous work, control polyurethane SMP foams degrade in 20% H_2_O_2_ over 9–10 days, providing a point of comparison for this study [37]. According to the relevant research reports, the control foam degrades completely in ~72 days in in vitro ‘real-time’ oxidative media of 3% H_2_O_2_. From these related studies, we can predict that PA foams can maintain their structure for ~115–140 days in real-time in vitro conditions.

When comparing real-time in vitro with in vivo degradation, the control PU foam degrades twice as fast in vitro in 3% H_2_O_2_ as in vivo when implanted in an aneurysm site [49,52,53,54]. By analogy, the lifespan of PA foams may be extended to 230–280 days in vivo. Under the physiological conditions of wounds, elevated ROS generated by inflammatory cells induce oxidative degradation of implanted biomaterials. The ability to control the rate of oxidative degradation in biomaterials can extend potential applications to longer-term implants. The addition of PAs provides a new tool for tuning SMP foam degradation that could be extended to other oxidatively degradable polymers [55], such as polylactic acid (PLA) [56], polyvinyl alcohol (PVA) [57,58], polycaprolactone (PCL) [59], polyvinylpyrrolidone (PVP) [60,61], poly(*N*-vinylcaprolactam) (PVCL) [62], and polyethylene glycol (PEG) [63,64].

Ideally, PA-foam-based wound dressings would remain as stable scaffolds in the wound with minimal degradation byproduct generation during use. At the same time, it is desired to provide a continuous release of PAs with antioxidant and antimicrobial properties to improve healing outcomes. Previous research shows that degradable polymer drug carriers can lead to burst release of drugs upon scaffold breakdown, resulting in an instantaneously high concentration of drug that may cause harm to the patient. In contrast, stable and sustained drug release from a biostable scaffold enables maintenance of the bioactive agent (i.e., PAs) at therapeutic concentrations.

#### 3.1.2. Microscopic Analysis

**Figure 2:**
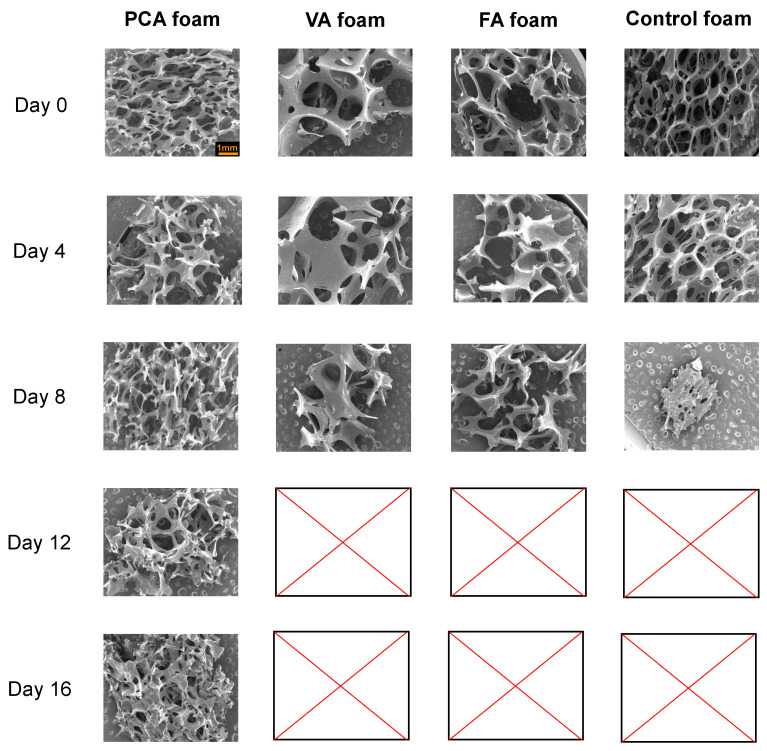
Scanning electron microscopic analysis of SMP foams over 16 days of degradation in in 20% H_2_O_2_. Blank pictures indicate that samples were completely degraded and could not be imaged. Scale bar applies to all images.

Micrographs of pore structures of SMP foams up to 16 days in accelerated oxidative media are shown in Figure 2. SEM images of all foams during the first four days of oxidative degradation show complete pore structure with minimal pore collapse or strut breakage. The mass remaining of all foams was >75% at this point, corroborating the microscopic analysis with the gravimetric analysis. The pore structure of the PCA foam was relatively stable over the full 16 days, with visible pores still present at day 16. Based on microscopic analysis, the PCA foam shows the best oxidative stability in 20% H_2_O_2_, which was supported by the gravimetric results. The VA and FA foams maintained pore structures until the 8th day of oxidative degradation. In contrast, only some fragments of control foams were visible in the SEM image on day 8, with evidence of complete pore collapse that primarily occurred between days 4 and 8. It should be noted that microscopic analysis was carried out on separate samples from the gravimetric analysis, and the microscopic analysis samples were cut at each time point. Thus, although there was mass remaining in the FA foam at 12 and 16 days in Figure 1, the cut microscopic analysis sample had completed degraded by this time.

PU SMP foams are more susceptible to oxidative degradation because of their porous structure. The surface area of porous foams is higher than that of non-porous films, and this porous structure increases the permeability of water and oxidizing agents into the scaffold [49]. Previous work showed that physical incorporation of antioxidant microparticles into PU SMP foams increases the pore stability and foam integrity during oxidative degradation [37]. Here, PAs were chemically incorporated into the PU SMP foam network, which improved the oxidative stability of the pore structure to maintain foam architecture over longer time frames. This effect may be beneficial for tissue scaffolding applications by increasing blood and tissue permeability of the foams, thereby enabling wound cell migration during healing [65].

#### 3.1.3. Thermal Characterization

**Figure 3:**
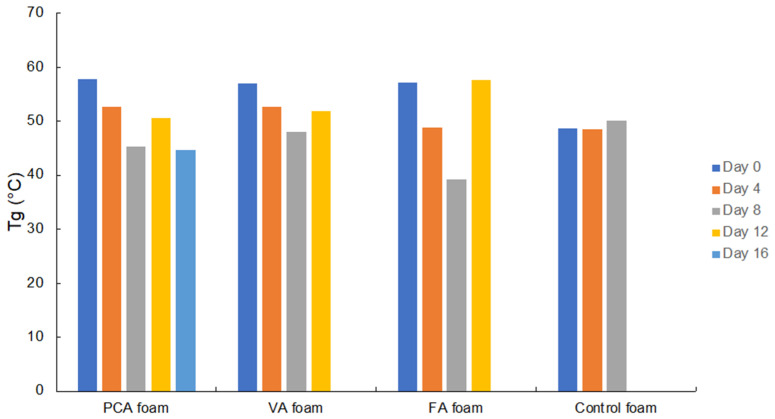
Thermal characterization of glass transition temperature (Tg) of SMP foams over 16 days of degradation in 20% H_2_O_2_. Tg’s were determined using differential scanning calorimetry (DSC).

The thermal analysis of SMP foams during accelerated oxidative degradation in 20% H_2_O_2_ was measured using DSC, Figure 3. For use as a wound dressing, we envision preheating SMP foam cylinders to above their dry T_g_ in an oven and radially compressing them before cooling. As long as the foams remain dry and the ambient temperature is below the dry T_g_, the SMP foams can maintain their compressed size for long storage times. In our previous work, we characterized PA foams in terms of shape memory properties in aqueous conditions [48]. Water penetrates into foams and breaks hydrogen bonds in the polyurethane network to decrease physical crosslinks and ultimately reduce T_g_ to below body temperature. In our thermal characterization and volume recovery testing, the wet T_g_ of all PA foams was below 25 °C, and they exhibited quick volumetric recovery (within 2 min) from their compressed, temporary shape back to their original, permanent shape in 37 °C water [48]. Thus, the high dry T_g_ enables stable storage and the reduced wet T_g_ enables shape recovery after implantation into the aqueous body environment.

In terms of degradation, T_g_ provides an indication of scaffold crosslink density over time (i.e., T_g_ is reduced as crosslink density decreases), which correlates with bulk degradation of the polymer network. The T_g_ of all tested PU SMP foams was fairly stable throughout degradation, which indicates that oxidation occurred primarily on the surface of the foams [66]. The T_g_ of the control foam remained almost unchanged during oxidative degradation. The T_g_ of PA foams generally decreased or remained the same during degradation. The higher initial (Day 0) T_g_ of the PA foams in comparison with the control foam (~57 °C vs. ~48 °C for control) is attributed to the phenolic rings that increase the backbone stiffness. Therefore, as the PAs were gradually released during degradation, the flexibility of the PU network increased, and the T_g_ was slightly reduced [67]. Surface degradation of PA SMP foams is beneficial to the delivery of PAs in the wound [68]. PAs would be continuously and slowly released from the PU foam after implantation, thus reducing toxicity and increasing the duration of their antioxidant effects to slow degradation, reduce inflammation, scavenge ROS, and promote wound healing.

#### 3.1.4. Spectroscopic Characterization and PA Delivery

**Figure 4:**
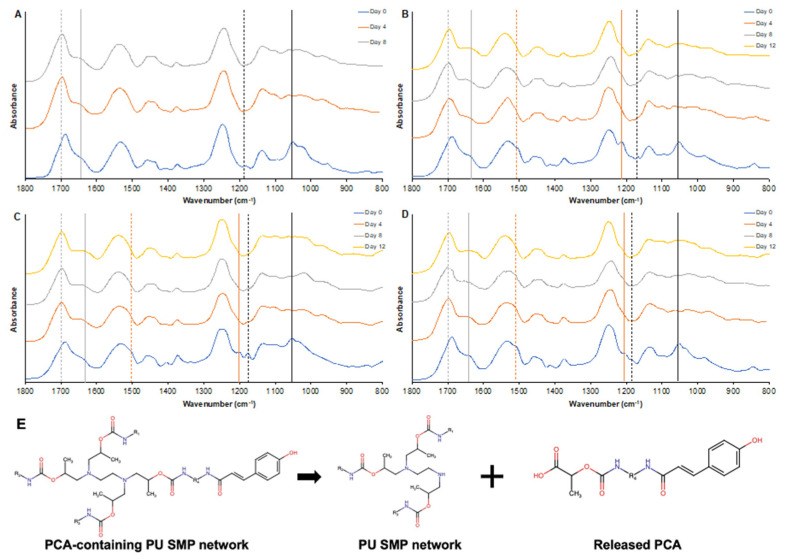
Spectroscopic analysis of (**A**) control, (**B**) PCA, (**C**) VA, and (**D**) FA SMP foams in 20% H_2_O_2_ over 12 days via ATR-FTIR. The grey dashed line shows the urethane C=O peak shift from 1680 cm^−1^ to 1690 cm^−1^ and the grey solid line shows the urea C=O peak at 1636 cm^−1^. The black dashed line at 1165 cm^−1^ and the black solid line at 1050 cm^−1^ shows the tertiary amine peak. The orange dashed line shows the C=C of the phenolic ring at 1500 cm^−1^ and the orange solid line shows the C–CH of the phenolic ring at 1180 cm^−1^. (**E**) Schematic of the scission of the tertiary amines of HPED leading the release of incorporated PCA from the PCA foam network in the accelerated degraded process. VA and FA share a similar release process from PU foams.

ATR-FTIR was utilized to spectroscopically characterize SMP foam surface chemistry throughout degradation in 20% H_2_O_2_, Figure 4. Figure 4A shows the standard spectroscopic changes that control PU foams undergo during oxidative degradation. The urethane peak shifts from 1680 cm^−1^ to 1690 cm^−1^ between days 0 and 4 (grey dashed line) relative to the urea peak at 1636 cm^−1^ (grey solid line). This change has been previously attributed to scission of the C-N bond of the tertiary amines in the polyols [49,69,70], which can be viewed by the reduction in the tertiary amine peaks at 1165 cm^−1^ (black dashed line) and 1050 cm^−1^ [49,69,70]. Other new peaks emerge below 1050 cm^−1^, which are attributed to secondary amine, aldehyde, and/or carboxylic acid formation [49,69,70].

The surface chemistry spectra of PCA, VA, and FA foams at day 0 show two strong peaks from the phenolic ring of the PAs in Figure 4B–D at 1500 cm^−1^ (orange dashed line) and 1180 cm^−1^ (orange solid line). PCA contains more C-CH_3_ groups than VA and FA, so the peak of PCA is stronger than that of VA and FA on Day 0. These two peaks decrease over time of degradation due to PA release from the foam. These data indicate that the PAs are released from the foams in the first four days of the accelerated degradation process due to the scission of the C-N bond of tertiary amines from polyols that are adjacent to PA-functionalized HDI groups, Figure 4E. These released PAs scavenge H_2_O_2_ and help to slow down the rate of oxidative degradation. Thereafter, the PAs were continuously released into the 20% H_2_O_2_ solution to increase the oxidative stability of the SMP foams. The results of the gravimetric and microscopic analyses also support this hypothesis. In summary, antioxidant PAs were delivered from PA foams to slow the oxidative degradation rate of polyurethane foams. PA-containing SMP foams that are implanted into wounds would be degraded by ROS from inflammatory cells, which could enable continuous slow release of PAs to remove ROS from the wound and promote healing.

### 3.2. PA Release from Foams

**Figure 5:**
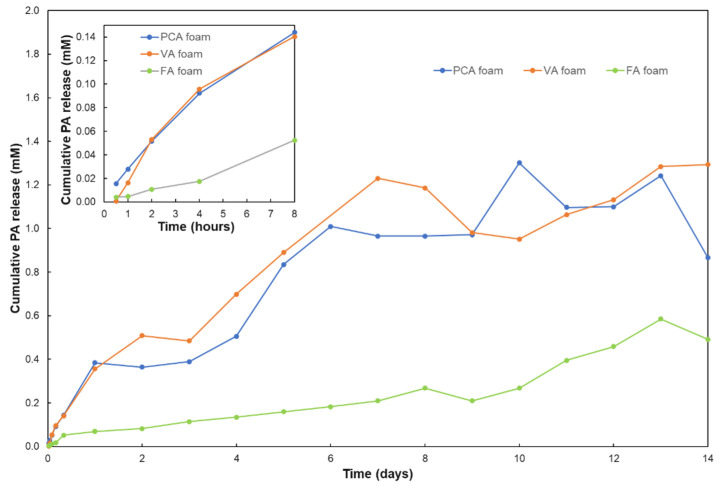
Analysis of cumulative PA release from SMP foams in PBS over 14 days. PA concentrations were determined using ultraviolet–visible (UV–Vis) spectrophotometry.

We determined the amount of PA released from each PA foam by measuring the absorbance values of key PA peaks in PBS according to the Beer–Lambert law. Measurable amounts of PCA, VA and FA were released from PA foams within 30 min, Figure 5. After that, the PAs continued to be released from the SMP foams over the two weeks of testing. Within 24 h, PCA and VA were released at relatively fast rates of 0.38 and 0.36 mM. PCA and VA have similar release rates, but the release rate of FA is slower. This result may be attributed to the molar masses of the PAs; PCA and VA are the same size, whereas FA is larger, thus slowing its diffusion out of the foam network. Although the release rate of FA was slower than that of PCA and VA, FA was continuously at an almost linear rate. These release studies were characterized in PBS, which does not degrade the SMP foams [49,53,54]. Therefore, we assume that PA release is accelerated in 20% H_2_O_2_ during degradation testing. Upon rapid release of PAs, they can scavenge ROS and slow down oxidative degradation.

The successful release of PAs underscores the potential of PA foams as wound dressings. Namely, PA foams could rapidly release PAs within 30 min to kill bacteria and promote blood clotting. PAs would then be released continuously at low levels over time to ensure wound sterility and reduce ROS in the wound to promote healing. The release mechanism of PAs from the foams into the PBS solutions in a short time is not clear to us, since they are theoretically chemically incorporated into foams by reactions between carboxylic acids and isocyanates, resulting in stable amide bonds. We hypothesize that some portion of each PA is not fully reacted into the PU network and is instead stabilized by secondary interactions, such as hydrogen bonding with urethanes or π–π interactions between phenolic rings. These physically incorporated PAs are released quickly, while those that are chemically incorporated will be released more slowly upon degradation of the foam network.

### 3.3. Cellular Antioxidant Activity

DCFDA/H_2_DCFDA was used as a cellular antioxidant activity assay with Caco-2 cells exposed to H_2_O_2_ and ABAP solutions after incubation with SMP foams for 1 h.

#### 3.3.1. CAA after Hydrogen Peroxide Exposure

**Figure 6:**
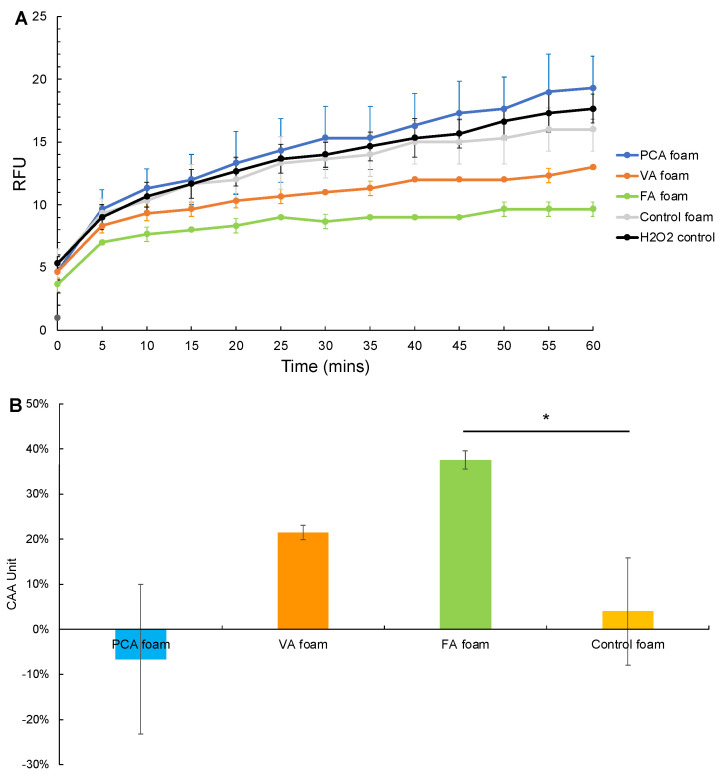
Cellular antioxidant analysis after PA foam and control foam incubation in H_2_O_2_. (**A**) H_2_O_2_-induced oxidation of DCFDA to DCF within Caco-2 cells over 60 min after incubation with SMP foams, (**B**) Cellular antioxidant activity (CAA). *n* = 3, mean ± standard deviation displayed, * *p* < 0.05 between H_2_O_2_ exposed to FA foam and control foam.

Figure 6A shows the kinetics of DCFDA oxidation in Caco-2 cells over 1 h of incubation in 0.01% H_2_O_2_ that had previously been exposed to SMP foams. The increase in fluorescence from fluorescent DCF formation following ROS oxidization of DCFDA in the cells was measured as an indication of cellular antioxidant activity. Thus, higher fluorescence intensity correlates with higher ROS levels within Caco-2 cells, which indicates lower antioxidant capabilities of the tested materials. The fluorescence curves of VA and FA foams exhibited a lower slope than that of the control foam and the H_2_O_2_ solution control. These smaller fluorescence curves show effective scavenging of cellular ROS by FA and VA foams. The corollary CAA values of FA and VA foams were 38% and 22%, respectively, which are higher than that of the control foam at 4%, Figure 6B. This result shows an increase in ROS scavenging by 5–10 fold with the inclusion of FA and VA into SMP foams. However, PCA foams had higher slopes than controls, resulting in an increase in cellular ROS levels and a slight, non-significant decrease in CAA.

#### 3.3.2. CAA after ABAP Exposure

**Figure 7:**
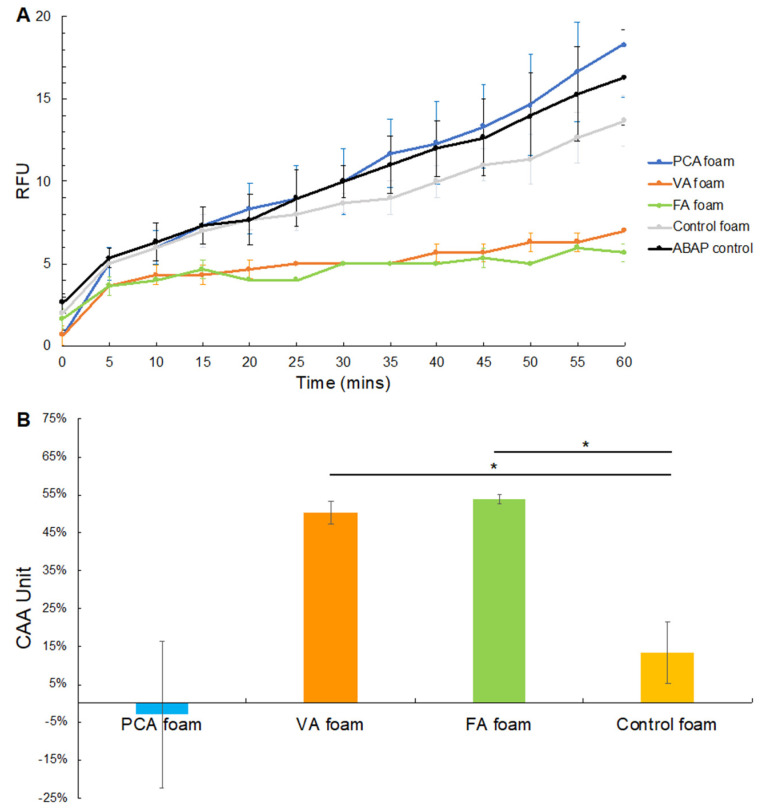
Cellular antioxidant analysis after PA foams and control foam incubation in ABAP. (**A**) Peroxyl radical-induced oxidation of DCFDA to DCF within Caco-2 cells over 60 min after incubation of ABAP with SMP foams, (**B**) Cellular antioxidant activity (CAA). *n* = 3, mean ± standard deviation displayed * *p* < 0.05 between H_2_O_2_ exposed to PA foams and control foam.

Figure 7A shows the kinetics of peroxyl radical generation due to DCFDA oxidation by ABAP over 1 h after ABAP incubation with SMP foams. ABAP is a ROS generator that forms peroxyl radicals within Caco-2 cells. The fluorescence curves of solutions that had been incubated with VA and FA foams exhibited a plateau after 5–10 min with lower fluorescence values compared to the control foam and ABAP solution control. The CAA values in ABAP solutions after exposure to VA and FA foams were 50% and 54%, respectively. These CAA values were ~4 times higher than that of the control foam, Figure 7B. Similar to the H_2_O_2_ study, PCA foams slightly reduced the CAA in comparison with control foams, indicating no antioxidant effects of PCA in the hour of foam incubation with ABAP.

In both studies, FA foams demonstrated the best effects on ROS scavenging to improve CAA. In our previous work, PAs with higher numbers of pendant -OH and -COC groups (e.g., FA) had higher antioxidant activity vs. PAs with fewer pendant groups (e.g., PCA) [33]. Natella et al. showed that the antioxidant efficacy of cinnamic acid (CA) derivatives was generally higher than that of benzoic acid (BA) equivalents [71]. FA is a derivative of CA, whereas VA is a derivative of BA. These structure property relationships were seen in H_2_O_2_ scavenging trends, where FA solutions exhibited higher H_2_O_2_ scavenging than VA and PCA solutions [33]. At a concentration of 0.08 mg/mL, FA scavenged 68% of H_2_O_2_, whereas VA and PCA scavenged 50% and 40%, respectively. These trends were also observed in the current work, showing that PAs can be rationally selected for incorporation into biomaterial scaffolds based on their properties in solution.

#### 3.3.3. Cytocompatibility

**Figure 8:**
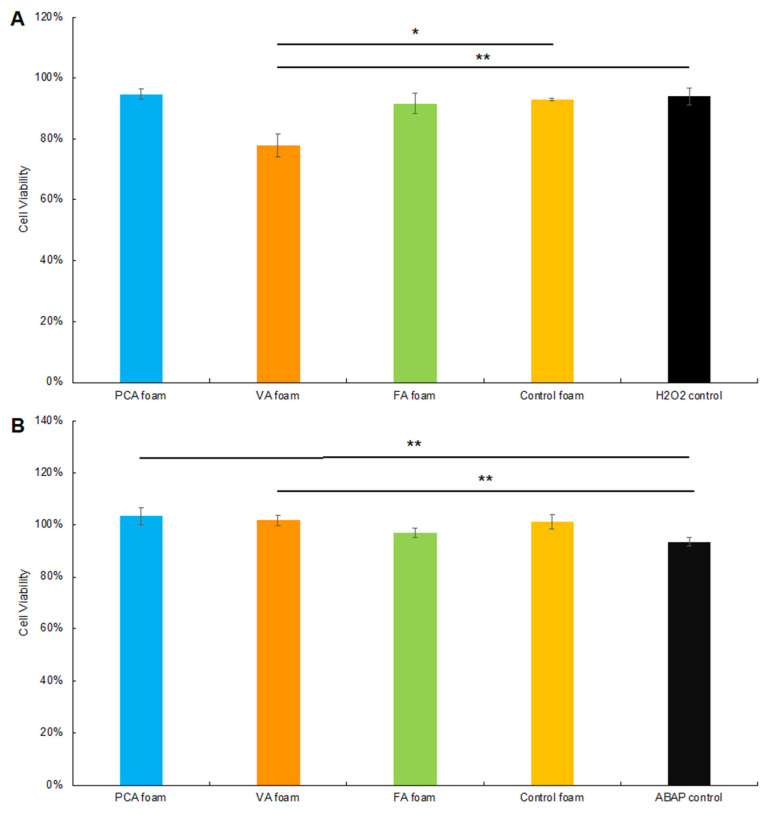
Caco-2 cell viability during the DCFDA/H2DCFDA assay process with SMP foams in (**A**) H_2_O_2_ and (**B**) ABAP. *n* = 3, mean ± standard deviation displayed, * *p* < 0.05 between PA foams and control foam, ** *p* < 0.05 between PA foams and ROS control.

To confirm that the ROS generator solutions that were incubated with SMP foams did not affect overall cell viability, Caco-2 cytocompatibility was measured using the Alamar blue assay, Figure 8. Cell viability was >78% after 2 h of incubation and >100% after 24 h of incubation with both 0.01% H_2_O_2_ and ABAP solutions that had been exposed to the SMP foam formulations. Thus, the reduced fluorescence intensity measured in Figure 6A and Figure 7A is due to intracellular ROS scavenging rather than apoptosis.

In summary, VA and FA foams enhanced cellular antioxidant ability with two types of ROS, H_2_O_2_ and peroxyl radicals from ABAP, whereas PCA foams did not affect the ROS levels within cells. Based on the PA release data presented in Section 3.1, VA and PCA are likely released from SMP foams more rapidly than FA. However, FA has higher antioxidant potency at lower concentrations, which may make it a better candidate for use in applications where ROS are of particular concern, such as in traumatic or chronic wounds. FA foams also slow oxidative degradation rates, indicating that FA may be the best PA choice for use in antioxidant scaffolds for both ROS scavenging and oxidative degradation resistance.

When we combine the data from these studies, we hypothesize that PAs must be released to provide effective antioxidant properties with surrounding cells. If VA and FA foams were used in a wound dressing, vanillic and ferulic acids may be continuously released into the wound to scavenge ROS and thus promote wound healing. In the short term (1 h), the PCA foam did not affect intracellular ROS levels. However, it is not clear whether this would be positive or negative for wound healing or what the longer-term effects may be as higher amounts of PCA are released at later time points. In general, the elevation of ROS within cells after indirect PCA foam exposure was moderate and did not lead to apoptosis. We aim to gain a better understanding of the long-term antioxidant effects of PCA foams in future studies.

In our previous study, PA-containing SMP foams showed excellent antimicrobial, cytocompatibility, hemocompatibility, and shape memory properties. The addition of PAs imparted the foams with antimicrobial properties against common wound pathogens, *Escherichia coli*, *Staphylococcus aureus* and *Staphylococcus epidermidis*. In this study, we further characterized PA foams to show that they improve oxidative degradation resistance and that FA and VA foams imparted intracellular ROS scavenging ability. This further validates the potential of PA foams as novel biomedical materials for wound dressings.

## 4. Conclusions

PCA foams demonstrated the best long-term oxidative degradation resistance in in vitro ROS-induced oxidative degradation testing. The oxidative degradation stability of PCA foams was increased by a factor of two compared with that of the control foam in accelerated oxidative conditions (20% H_2_O_2_). Similarly, the PCA foam maintained a stable pore structure throughout degradation. FA and VA foams also slow oxidative degradation rates in accelerated media. When we investigated the intracellular ROS scavenging ability of PA foams as an initial indication of their potential effect on wound healing, VA and FA foams showed better ROS scavenging ability in the CAA assay than PCA foams, with the highest CAA observed with FA foams. PCA foams induced a slight increase in intracellular ROS. We will further explore the effects of PCA and ROS on wound healing in subsequent experiments and expand the analysis of the antioxidant properties of biomaterials with incorporated PAs as potential wound healing platforms.

## Data Availability

The data presented in this study are contained with the article. Further details are available on request from the corresponding author.
